# Complete Genome Sequence of Sphingobium sp. Strain YG1, a Lignin Model Dimer-Metabolizing Bacterium Isolated from Sediment in Kagoshima Bay, Japan

**DOI:** 10.1128/genomeA.00267-18

**Published:** 2018-04-26

**Authors:** Yukari Ohta, Yasuhiro Shimane, Shinro Nishi, Junko Ichikawa, Kanako Kurosawa, Taishi Tsubouchi, Shun'ichi Ishii

**Affiliations:** a R&D Center for Marine Bioscience, Japan Agency for Marine-Earth Science and Technology (JAMSTEC), Yokosuka, Kanagawa, Japan; b Department of Bacteriology, Osaka City University Graduate School of Medicine, Osaka, Japan; c R&D Center for Submarine Resources, JAMSTEC, Nankoku, Kōchi, Japan

## Abstract

Sphingobium sp. strain YG1 is a lignin model dimer-metabolizing bacterium newly isolated from sediment in Kagoshima, Japan, at a depth of 102 m. Here, we report the complete genome nucleotide sequence of strain YG1.

## GENOME ANNOUNCEMENT

Lignin is one of the most abundant biomass compounds and is composed of complex polyaromatic structures (1). Only a small number of bacteria belonging to Sphingobium and closely related genera within the family Sphingomonadaceae have been reported to specifically cleave the chemical linkages between lignin monoaromatic units (2–5), whereas numerous isolates within the family Sphingomonadaceae showed degradation of aromatic monomers by using diverse gene sets for aromatic-ring cleavage which are important for the detoxification of persistent aromatic hydrocarbons (6, 7).

It has been demonstrated that the specific cleavage of the main linkages between lignin monomeric units proceeds via a combination of nonradical enzymes for these isolates (2–5). To gain deeper insight into the diversity, distribution, and evolution of the genes for metabolizing lignin-related low-molecular-weight aromatics, we screened new Sphingobium strains using guaiacylglycerol-β-guaiacyl ether (GGGE) as a model dimeric substrate having a β-ether linkage that mimics the major substructure in lignin. Then, we successfully isolated a GGGE-metabolizing strain from sediment in Kagoshima Bay at a depth of 102 m (31.7N, 130.8E) and designated it strain YG1.

Total genomic DNA of strain YG1 was extracted using a NucleoSpin plant II midi kit (TaKaRa Bio), according to the manufacturer’s protocol. Whole-genome sequencing of strain YG1 was performed using Pacific Biosciences (PacBio) RS II sequencers (8). PacBio reads totaling 5,563,895 bases were obtained using SMRT Analysis (version 2.3.0) and assembled into four circular contigs using the Hierarchical Genome Assembly Process version 3.0 (HGAP 3.0) assembler (9).

The genome of strain YG1 is composed of two circular chromosomes (3,435,856 and 1,847,201 bases, with redundancies of 159- and 196-fold, respectively), and two plasmids (214,196 and 66,642 bases, with redundancies of 98- and 59-fold, respectively). The G+C contents of the contigs are 63.8, 62.1, 61.9, and 58.5%. We identified 4,992 protein-coding sequences (CDSs) using the MetaGene Annotator (10). We annotated the predicted CDSs through a BLAST+ search against the NCBI nonredundant protein sequences (11).

We predicted overall metabolic physiological functions of strain YG1 using MAPLE (version 2.3.0) with bidirectional best-hit matches (12, 13). Through orthologous analysis using the KEGG Orthology (KO) database, we bioinformatically found a LigF-type β-etherase gene and a gene island that encodes seven enzymes for converting 3,4-dihydroxybenzoate to pyruvate in the general pathway of benzoate degradation by aerobic bacteria on the second chromosome. All the above-mentioned genes are known to be involved in the GGGE degradation pathway in Sphingomonas paucimobilis SYK-6 (2). Thus, our study provides genetic information for the lignin metabolism of the newly isolated Sphingobium sp. strain YG1.

### Accession number(s).

This whole-genome shotgun project has been deposited in DDBJ/ENA/GenBank under the accession no. AP018518 to AP018521.
